# Randomised controlled trial comparing the clinical and cost-effectiveness of various washout policies versus no washout policy in preventing catheter associated complications in adults living with long-term catheters: study protocol for the CATHETER II study

**DOI:** 10.1186/s13063-022-06577-2

**Published:** 2022-08-04

**Authors:** Mohamed Abdel-fattah, Diana Johnson, Lynda Constable, Ruth Thomas, Seonaidh Cotton, Sheela Tripathee, David Cooper, Sue Boran, Konstantinos Dimitropoulos, Suzanne Evans, Paraskeve Granitsiotis, Hashim Hashim, Mary Kilonzo, James Larcombe, Paul Little, Sara MacLennan, Peter Murchie, Phyo Kyaw Myint, James N’Dow, John Norrie, Muhammad Imran Omar, Catherine Paterson, Graham Scotland, Nikesh Thiruchelvam, Graeme MacLennan

**Affiliations:** 1 Aberdeen Centre for Women’s Health Research, University of Aberdeen, Aberdeen, UK; 2 Centre for Healthcare Randomised Trials, University of Aberdeen, Aberdeen, UK; 3 Academic Urology Unit, University of Aberdeen, Aberdeen, UK; 4 Health Services Research Unit, University of Aberdeen, Aberdeen, UK; 5 The Queen’s Nursing Institute, London, UK; 6 Bladder Health UK, Birmingham, UK; 7 Lothian Health Board, Edinburgh, UK; 8 North Bristol NHS Trust, Bristol, UK; 9 Health Economics Research Unit, Institute of Applied Health Sciences, University of Aberdeen, Aberdeen, UK; 10 JMed Limited, Sedgefield, UK; 11 Primary Care Research Centre, University of Southampton, Southampton, UK; 12 Academic Primary Care Research Group, University of Aberdeen, Aberdeen, UK; 13 Ageing Clinical & Experimental Research Team, University of Aberdeen, Aberdeen, UK; 14 Usher Institute, University of Edinburgh, Edinburgh, UK; 15 School of Nursing, Midwifery and Public Health, University of Canberra, Canberra, Australia; 16 Cambridge University Hospitals NHS Foundation Trust, Cambridge, UK

**Keywords:** Catheter blockage, Catheter washout solutions, Catheter maintenance solutions, Indwelling catheter, Long-term catheter, Symptomatic catheter-associated urinary tract infection

## Abstract

**Background:**

Various washout policies are widely used in adults living with long-term catheters (LTC). There is currently insufficient evidence on the benefits and potential harms of prophylactic LTC washout policies in the prevention of blockages and other LTC-related adverse events, such as urinary tract infections. CATHETER II tests the hypothesis that weekly prophylactic LTC washouts (normal saline or citric acid) in addition to standard LTC care reduce the incidence of catheter blockage requiring intervention compared to standard LTC care only in adults living with LTC.

**Methods:**

CATHETER II is a pragmatic three-arm open multi-centre superiority randomised controlled trial with an internal pilot, economic analysis, and embedded qualitative study. Eligible participants are adults aged ≥ 18 years, who have had a LTC in use for ≥ 28 days, have no plans to discontinue the use of the catheter, are able to undertake the catheter washouts, and complete trial documentation or have a carer able to help them. Participants are identified from general practitioner practices, secondary/tertiary care, community healthcare, care homes, and via public advertising strategies. Participants are randomised 1:1:1 to receive a weekly saline (0.9%) washout in addition to standard LTC care, a weekly citric acid (3.23%) washout in addition to standard LTC care or standard LTC care only. Participants and/or carers will receive training to administer the washouts. Patient-reported outcomes are collected at baseline and for 24 months post-randomisation. The primary clinical outcome is catheter blockage requiring intervention up to 24 months post-randomisation expressed per 1000 catheter days. Secondary outcomes include symptomatic catheter-associated urinary tract infection requiring antibiotics, catheter change, adverse events, NHS/ healthcare use, and impact on quality of life.

**Discussion:**

This study will guide treatment decision-making and clinical practice guidelines regarding the effectiveness of various prophylactic catheter washout policies in men and women living with LTC. This research has received ethical approval from Wales Research Ethics Committee 6 (19/WA/0015).

**Trial registration:**

ISRCTN ISRCTN17116445. Registered prospectively on 06 November 2019

**Supplementary Information:**

The online version contains supplementary material available at 10.1186/s13063-022-06577-2.

## Administrative information

Note: The numbers in curly brackets in this protocol refer to SPIRIT checklist item numbers. The order of the items has been modified to group similar items (see http://www.equator-network.org/reporting-guidelines/spirit-2013-statement-defining-standard-protocol-items-for-clinical-trials/).

**Table:** 

Title {1}	The CATHETER II Study: Randomised Controlled Trial CompAring THE Clinical And CosT-Effectiveness Of VaRious Washout Policies Versus No Washout Policy In Preventing Catheter Associated Complications In Adults Living With Long-Term Catheters
Trial registration {2a and 2b}.	ISRCTN (ISRCTN17116445). Registered prospectively on 06 November 2019.
Protocol version {3}	Version 10, 07 March 2022
Funding {4}	National Institute for Health Research (NIHR) Health Technology Assessment (HTA) Programme (project number 17/30/02).
Author details {5a}	^1^Aberdeen Centre for Women’s Health Research, University of Aberdeen, Aberdeen, UK. ^2^Centre for Healthcare Randomised Trials, University of Aberdeen, Aberdeen, UK. ^3^Academic Urology Unit, University of Aberdeen, Aberdeen, UK. ^4^Health Services Research Unit, University of Aberdeen, Aberdeen, UK. ^5^The Queen’s Nursing Institute, London, UK. ^6^Bladder Health UK, Birmingham, UK. ^7^Lothian Health Board, Edinburgh, UK. ^8^North Bristol NHS Trust, Bristol, UK. ^9^Health Economics Research Unit, Institute of Applied Health Sciences, University of Aberdeen, Aberdeen, UK. ^10^JMed Limited, Sedgefield, UK. ^11^Primary Care Research Centre, University of Southampton, Southampton, UK. ^12^Academic Primary Care Research Group, University of Aberdeen, Aberdeen, UK. ^13^Ageing Clinical & Experimental Research Team, University of Aberdeen, Aberdeen, UK. ^14^Usher Institute, University of Edinburgh, Edinburgh, UK. ^15^School of Nursing, Midwifery and Public Health, University of Canberra, Canberra, Australia. ^16^Cambridge University Hospitals NHS Foundation Trust, Cambridge, UK.
Name and contact information for the trial sponsor {5b}	**Co-sponsor 1.** University of Aberdeen Research Governance Office, Room 1.126, Polwarth Foresterhill, Aberdeen, AB25 2ZD researchgovernance@abdn.ac.uk **Co-sponsor 2.** Grampian Health Board Research and Development Office, Foresterhill House Annexe Foresterhill, Aberdeen, AB25 2ZB gram.randd@nhs.scot
Role of sponsor {5c}	The sponsor has no role in the study design; collection, management, analysis or interpretation of the data; and the writing or submission of reports for publication.

## Introduction

### Background and rationale {6a}

Long-term catheters (LTC) are used by patients with conditions such as intractable urinary incontinence or chronic urinary retention to empty the bladder. Chronic urinary retention can be secondary to a variety of conditions such as enlarged prostate, underactive bladder, and neurological conditions such as spinal cord injury and multiple sclerosis [1, 2]. The National Institute for Health and Care Excellence (NICE) CG139 recommends an indwelling catheter for those who are unable to perform intermittent catheterisation or those for whom toileting is difficult [3]. The indwelling catheter may be inserted into the urinary bladder via the urethra (urethral catheter) or via the anterior abdominal wall (suprapubic catheter). The urine is either drained into a catheter bag or emptied when convenient with the use of a catheter valve. Current NHS standard care includes a change of the catheter bag or valve every week by the patient, or carer, and a change of the catheter every 4–12 weeks by the clinical team [4].

There is no robust evidence to support a definition for the duration of catheter stay that constitutes “long-term” catheter use. Evidence from a Cochrane review [2] indicate that most studies defined LTC use as urethral or supra-pubic catheter in situ for > 28 days with predicted use over 6–12 months. This definition was also used in the NICE CG139 [3]. LTC use may be for many years, Wilde et al. [5] reported a mean duration of six years in 202 participants (median 3.25 years).

The exact prevalence of LTC use is not known. It was estimated that between 0.2–0.5% of the general population of the United Kingdom (UK) are living with a LTC (unpublished data, Farrer B, Norris S, 2018; personal communication, NIHR Clinical Research Network, NHS Research Scotland Primary Care Network). New evidence estimates the prevalence in the UK at approximately 90,000 LTC users (or 0.14% of the overall population) [6]. It is anticipated that LTC use will continue to increase with the increasing ageing population [7].

LTC can be associated with several adverse events [5] which affect the daily life of patients and can consume substantial NHS resources [8]. Wilde et al. reported typical adverse events of LTC blockage (34% of participants): symptomatic catheter-associated urinary tract infections (S-CAUTI), whether or not this has been proven bacteriologically (57%); accidental dislodgment (28%); urinary leakage (67%); bladder spasms (59%); kinks/twists (42%) and pain (49%) [1].

LTC blockages often occur secondary to the formation of encrustations on the luminal and outer surfaces of the catheter, with an incidence of 40–50% in patients in most studies [7, 9–11]. Wilde et al. in 2017 [1] assessed 202 patients with LTC over 12 months and showed that 34% of patients reported blockage and a rate of 8.54/1000 days of catheter use. Catheter blockage is considered a medical emergency and can lead to distress, autonomic dysreflexia in patients with spinal cord injury at or above T6, increased healthcare utilisation and urosepsis [12]. Current best practice for the management of LTC blockage requires a regular (and more frequent) change of the catheter [3]. Catheter washouts are often used in these cases despite the lack of evidence on their benefit, potential harm, best solution to be used, appropriate volume and frequency, and individual impact on quality of life among patients with LTC [2].

Several catheter washouts policies are used in clinical practice for the prevention and/or management of LTC blockage. Washouts used are of different types (normal saline, acidic, antimicrobial), volumes and frequency of administration. The Cochrane review [2] assessed the best available evidence and concluded that there is insufficient evidence to determine whether prophylactic catheter washout policies had a beneficial or harmful effect on any of the outcomes in patients with LTC. The authors recommended a rigorous and methodologically robust randomised controlled trial to assess the clinical and cost-effectiveness of washout policies in patients with LTC.

Muncie et al. [13] compared saline washouts versus no washouts policy over 24 weeks in a limited population and showed no significant differences in S-CAUTI per 100 days of catheter use. Concerns exist that the use of washouts can damage the bladder mucosa and possibly increase the risk of S-CAUTI. NICE CG139 recommend that “[catheter] washouts must not be used to prevent catheter-associated infections” [3].

In CATHETER II, researchers will evaluate what is the clinical and cost-effectiveness, patient acceptability and satisfaction, and safety of weekly prophylactic catheter washout policies in addition to standard LTC care compared to standard LTC care only, in adults living with LTC.

### Objectives {7}

The aim of the study is to determine whether the addition of a policy of prophylactic weekly catheter washouts to current standard LTC care improves the outcome of care for people living with a LTC in the UK.

The hypotheses being tested are:

1. Does a policy of weekly prophylactic normal saline catheter washouts plus standard LTC care result in a relative reduction of 25% (or more) in catheter blockage requiring intervention compared to standard LTC care alone?
2. Does a policy of weekly prophylactic acidic catheter washouts plus standard LTC care result in a relative reduction of 25% (or more) in catheter blockage requiring intervention compared to standard LTC care alone?

### Trial design {8}

CATHETER II is a pragmatic three-arm, parallel-group, open multi-centre superiority randomised controlled trial. It compares the clinical and cost-effectiveness, patient acceptability and satisfaction, and safety of weekly prophylactic catheter washouts policies in addition to standard long-term catheter (LTC) care compared to standard LTC care only, in adults living with LTC.

## Methods: participants, interventions and outcomes

### Study setting {9}

Participants are recruited from general practitioner (GP) practices, secondary and tertiary care hospitals, community hospitals and care homes including nursing homes in Scotland, England and Wales. Participants are also recruited via a public advertising strategy such as on websites and social media platforms, utilising targeted advertisements. A list of current study sites can be obtained from the CATHETER II study website [14].

### Eligibility criteria {10}

The inclusion criteria are:

- Aged ≥ 18 years
- Catheter has been in use for ≥ 28 days
- No plan for discontinuation of LTC at the time of recruitment
- Able to undertake catheter washouts or has a designated person (relative, friend, other informal carer or paid/NHS healthcare worker) able to perform washouts
- Able to complete the trial documentation or has a designated person able to assist with trial documentation
- Any type and route of LTC can be included

The exclusion criteria are:

- Intermittent self-catheterisation
- Pregnant or contemplating pregnancy
- Spinal cord injury at or above the sixth thoracic vertebra (T6) (risk of autonomic dysreflexia)
- Ongoing S-CAUTI (until treatment is complete)
- Visible haematuria (unless investigated/ treated)
- Known allergies to either of the catheter washout solutions
- Current bladder cancer (until treatment is complete and patient discharged from cancer surveillance programme)
- Known bladder stones (until treatment is complete)
- Unable to provide consent due to incapacity
- Any other clinical and social reasons that would be deemed by the recruitment team to be unsuitable for the study

### Who will take informed consent? {26a}

Written informed consent from participants is sought and obtained by delegated Good Clinical Practice (GCP) trained members of the local research team. Consent may be sought during a face-to-face appointment, or participants may have the consent discussion with a member of the research team by telephone, and then return their completed consent form by post for countersignature by the member of the team who had had the consent discussion with the participant. If a potential participant with the capacity to consent is unable to provide written consent due to a physical incapacity, an impartial witness will witness the oral consent process and sign the study consent form on the participant’s behalf. If the participant requires help from a designated person (relative, friend, other informal carers) to carry out the washouts or to assist with trial documentation, written consent is sought from the designated person for the relevant activities by a GCP-trained member of the local research team. Written or verbal consent to participate in the qualitative interview study is obtained separately from participants and healthcare professionals by a GCP-trained qualitative researcher. All consent is taken in accordance with the GCP guidelines.

### Additional consent provisions for collection and use of participant data and biological specimens {26b}

Participants can opt in to be contacted about participating in future relevant research. No biological specimens are collected in CATHETER II.

### Interventions

#### Explanation for the choice of comparators {6b}

Uro-Tainer® Twin SUBY G and Uro-Tainer® NaCl 0.9% CE 100 ml are the most commonly used catheter washout solutions in the UK (correspondence, B.Braun Medical AG) and have been provided gratis for the study by the manufacturer. We are comparing these against standard LTC care (i.e. with no planned prophylactic LTC washouts).

#### Intervention description {11a}

The interventions being compared are:

- *Intervention arm (A):* Saline washouts. A policy of weekly prophylactic normal saline catheter washouts plus standard LTC care. One application of 100 ml 0.9% NaCl per washout (Uro-Tainer® NaCl 0.9% CE)
- *Intervention arm (B):* Acidic washouts. A policy of weekly prophylactic acidic catheter washouts plus standard LTC care. Two sequential applications of 30 ml 3.23% citric acid per washout (Uro-Tainer® Twin SUBY G)
- *Control arm (C):* Standard LTC care only with no prophylactic catheter washout

Washouts are administered in accordance with best practice technique at the time of the regular weekly catheter bag or valve change, to reduce the risk of introducing infection by minimising the breakage of the closed drainage system. Participants and/or their relatives, friends, or other informal carers will receive training to administer catheter washouts from an appropriately trained member of the local study team to enable them to self-administer the washouts in accordance with best practice. Training is provided with either face-to-face or by video/phone consultation and is supported with an online training video and hardcopy instructions for use with troubleshooting advice. If a health professional usually changes the catheter bag or valve for a participant and capacity and capability allows, they will be asked to undertake training and perform the washout within the study. The washouts are couriered directly to the participant from the trial office.

#### Criteria for discontinuing or modifying allocated interventions {11b}

Washout use is discontinued if a participant stops using the LTC for 28 days or longer, or if they no longer wish to carry out regular washouts, or if they are unable to carry out the washouts following training, or if they withdraw consent for the monthly data collection schedule and decline for this information to be collected less frequently. Where deemed clinically necessary by the clinical team, the pragmatic design of the study permits the following changes to washout policies:

- An increase in the frequency of LTC washouts, at the onset of the study or following regular review during the course of the study
- A change in the type of washout, at the onset of the study or following regular review during the course of the study
- The use of prophylactic washouts in the control arm, following regular review during the course of the study (but not at the onset of the study)

#### Strategies to improve adherence to interventions {11c}

The site team ask the participant about adherence at every monthly contact, and this is recorded in the case report form (CRF). We would expect at least 80% of participants to be undertaking 60% of their washouts and this is monitored during the internal pilot phase. If the threshold is not met, we may consider adapting or offering participants more training sessions.

#### Relevant concomitant care permitted or prohibited during the trial {11d}

Standard catheter care is permitted during the trial and managed by the participant’s usual health care team. The use of prophylactic washout solutions in the standard care arm is discouraged at the onset of the study. There is no change to other care received by the participant.

#### Provisions for post-trial care {30}

At the conclusion of the study, no further washout solutions will be sent to participants by the trial team, and they will continue their care and treatment in line with standard NHS clinical care.

### Outcomes {12}

The primary clinical outcome is catheter blockage requiring intervention up to 24 months post-randomisation expressed as number per 1000 catheter days.

Intervention is defined as any of the following: unplanned catheter removal or change or washout performed by the participant/designated person or required unplanned visits to/from any healthcare provider, or hospital admission.

The primary economic outcome is the incremental cost per quality-adjusted life year gained for each washout policy compared to standard LTC care only.

Secondary outcomes include:

- S-CAUTI requiring antibiotics use (as defined by Pickard et al. [15])
- Duration of LTC in use, catheter change due to other reasons than blockage
- Adverse events;
- Hospital admissions, GP/nurse outpatient visits for catheter-related complications
- Generic quality of life as assessed by EQ-5D-5L [16] (EuroQol Questionnaire – 5 Dimensions – 5 Levels)
- Condition-specific quality of life assessed by ICIQ-LTCqol [17] (International Consultation on Incontinence Modular Questionnaire – Long Term Catheter quality of life)
- Adherence to allocated interventions
- Patients’ convenience and satisfaction assessed by an adapted version of the abbreviated Treatment Satisfaction Questionnaire for medication [18]
- Impact on day-to-day activities using the General Self-Efficacy Scale (GSE) [19] and ICECAP-A (ICEpop CAPability measure for Adults) (≤ 65 years) or ICECAP-O [20] (ICEpop CAPability measure for Older people) > 65 years
- Time and travel costs for patients and their relatives, friends or informal carers
- Discontinuation of catheter use
- Events changing the type and/or frequency (or cessation) of catheter washouts in arms A and B and rates of commencing on prophylactic washouts in arm C

Qualitative study outcomes:

- Participants’ experience of LTC-related adverse events such as blockage, S-CAUTI, urinary incontinence and bladder pain.
- Participants’ attitudes/preferences to washout versus no washout policies and expected outcomes (prior to randomisation or knowing their allocated study group) (acceptability).
- Participants’ experience with washout/no washout policies and evaluation of outcomes (satisfaction).
- Clinicians’ attitudes towards the influence of washout policies on outcomes.
- Participants’ and clinicians’ experience of training provided and enactment of the treatment skill. This would clarify the fidelity of the intervention.

### Participant timeline {13}

**Fig. 1:**
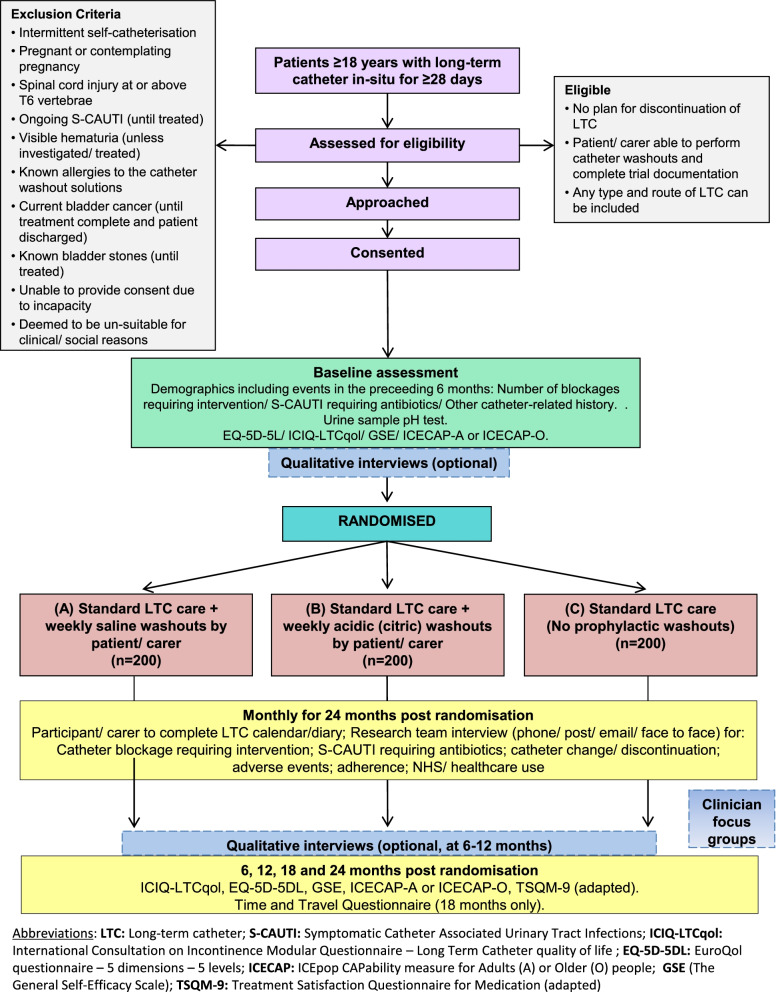
Flow diagram describing the participant timeline through CATHETER II

The flowchart in Fig. 1 describes the participant timeline throughout the study.

### Sample size {14}

We have used information from a survey of experts and patients and also from available literature to decide that for washouts to be worthwhile there must be a reduction in LTC blockage of 25% [1] (and personal communication, Cambridge PPI group survey). In our case, this would be a reduction in the rate of blockage from 11.8 per 1000 days (unpublished data, Farrer B, Norris S, 2018) to 8.9 per 1000 days. Participants will be followed up for 2 years. The trial has a 90% power and a significance level of 2.5%. The number of blockages has a negative binomial distribution with a dispersion parameter of 0.6. Recruiting 200 participants per arm allows for approximately 50 out of 730 loss to follow-up days. All available days of follow-up are to be used. The formula from Zhu and Lakkis [21] was used to calculate the sample size for comparing two negative binomial rates.

### Recruitment {15}

We are recruiting participants from GP practices, secondary and tertiary care hospitals, community hospitals and care homes including nursing homes. Recruitment strategies differ between sites depending on local geographic and NHS organisation factors. Potential participants may be identified by database searches; A&E or in urology, neurorehabilitation and care of the elderly outpatient clinics and wards; and from continence clinics and district nurse and community nurse teams. Potential participants are approached and provided with an invitation letter and a short patient information leaflet. A follow-up telephone call or reminder letter may be sent. In care homes, the care home manager identifies potentially eligible individuals who are approached and provided with a patient information leaflet. Across all settings, a study poster may be displayed as a resource to support recruitment.

Participants may also be recruited through research registries and advertising campaigns utilising methods such as social media, mainstream media, websites and newsletters and with the support of digital marketing agencies. Potential participants identified in this way are referred to a suitable local recruiting site, if one is available, or recruited centrally with all study activities delivered remotely and with clinical oversight by the chief investigator.

All participants are provided with a full patient information leaflet. Eligibility is confirmed and consent is taken prior to baseline data collection and randomisation.

If a participant requires help from a relative, friend, or another informal carer to administer the washouts or complete the patient questionnaires or catheter diary, the relative, friend, or another informal carer is also provided with a patient information leaflet and consented to the study.

An internal pilot with stop/go criteria is embedded to establish whether the projected recruitment rate is achievable.

### Assignment of interventions: allocation

#### Sequence generation {16a}

Participants are allocated 1:1:1 to one of the three trial arms by a member of the local research team using a centralised computerised randomisation system (administered by the Centre for Healthcare Randomised Trials (CHaRT), University of Aberdeen. Random allocation uses the minimisation covariates: region; gender; age (< 45 years, 45–64 years and ≥ 65 years); residential status (care home vs community); previous blockages requiring intervention in the last 6 months (0 vs ≥ 1); previous S-CAUTI requiring antibiotics in last 6 months (0 vs ≥ 1); Urine pH (normal vs acidic vs alkaline vs not available).

#### Concealment mechanism {16b}

The allocation sequence is concealed by the use of a centralised computerised randomisation system.

#### Implementation {16c}

Delegated site personnel will enrol participants on the study website in which the randomisation system is embedded. The centralised computerised randomisation system generates the allocation sequence and assigns the trial arm.

### Assignment of interventions: blinding

#### Who will be blinded {17a}

It is not possible to blind the allocated study arm.

#### Procedure for unblinding if needed {17b}

Not applicable, the allocated study arm is not blinded.

### Data collection and management

#### Plans for assessment and collection of outcomes {18a}

The source and timing of measures are summarised in Table 1. The baseline assessment can be done remotely or face-to-face. Participants complete the baseline questionnaire prior to randomisation, with the assistance of their relative, friend or informal carer or research team where required. A catheter urine sample for pH testing will obtained from all participants and tested immediately using the simple urine dipstick test (where participants are recruited without face-to-face contact, the dipstick test and instructions are sent to them by post). Alternatively, a historical urine pH measurement in the 3 months preceding randomisation may be collected from medical records. The local research team completes the baseline CRF.

**Table 1:** Source and timing of measures

**Measure**	**Source**	**Randomisation**				
		**Pre**^a^	**Post**			
Catheter blockage requiring intervention	D & CRF		Monthly completion for 24 months			
S-CAUTI requiring antibiotics						
Prophylactic antibiotic use						
Catheter change						
Adverse events						
NHS/healthcare use						
			**Months**			
			**6**	**12**	**18**	**24**
EQ-5D-5L	PQ	**✓**	**✓**	**✓**	**✓**	**✓**
ICIQ-LTCqol	PQ	**✓**	**✓**	**✓**	**✓**	**✓**
GSE Scale	PQ	**✓**	**✓**	**✓**	**✓**	**✓**
ICECAP-A or O	PQ	**✓**	**✓**	**✓**	**✓**	**✓**
Satisfaction with treatment	PQ	**✓**	**✓**	**✓**	**✓**	**✓**
Participant/relative, friend or informal carer’s time and travel	PQ				**✓**	

*CRF* case report form, *D *LTC diary/calendar, *PQ *participant/relative, friend or informal carer-completed questionnaire, *S-CAUTI *symptomatic catheter-associated urinary tract infection, *EQ-5D-5L *EuroQol questionnaire – 5 dimensions – 5 levels, *ICIQ-LTCqol *International Consultation on Incontinence Modular Questionnaire – Long Term Catheter quality of life, *GSE *General Self-Efficacy Scale, *ICECAP-A *ICEpop CAPability measure for Adults, *ICECAP-O* ICEpop CAPability measure for Older people

^a^Pre-randomisation is after informed consent has been given but prior to randomisation

Participants (or the relative, friend or informal carer carrying out the washout or research team) record LTC-related events on their LTC calendar/diary, adapted from a purpose-built diary that has been successfully used in a previous randomised controlled trial in this field [1].

A delegated member of the research team collects the primary outcome, several of the secondary outcomes, adverse events, and adherence approximately monthly for 24 months by telephone or other agreed methods from the participant and/or the relative, friend or informal carer.

Postal or web-based questionnaires are completed by participants with or without assistance from their relative, friend or informal carer or research team at 6, 12, 18, and 24 months after randomisation.

An embedded qualitative component is included to evaluate the participant’s experiences of LTC-related adverse events and their attitudes to, and experiences of, catheter washout (including training). Thirty to 40 participants will be interviewed pre-randomisation and 6–12 months into the study. Participants will be selected using purposive sampling to ensure the diverse characteristics of the population. Approximately twenty health care workers will take part in focus groups 6–12 months into the study to explore attitudes towards washout policies and views on likely outcomes.

#### Plans to promote participant retention and complete follow-up {18b}

Participant retention is promoted by regular monthly contact with the site team to collect outcome measures. Participants receive one reminder to complete each follow-up questionnaire. A small token of appreciation is sent to participants on receiving each completed follow-up questionnaire, unless they opt out on the study consent form. All data collected up to the point of complete withdrawal are retained and used in the analysis. Participants who do not complete their trial follow-up but for whom any outcome data are available are included in the study analysis. If a participant stops using a long-term catheter ≥ 28 days, all data collected up to the point of stopping long-term catheter use are retained and used in the analysis and they are requested to complete an exit questionnaire (EQ-5D-5L only). Deviations from the allocated study arm are recorded in the monthly CRFs and assessed as a secondary outcome measure.

#### Data management {19}

Local study team members as listed on the delegation log can enter locally collected data. Safety data, CRFs and participant questionnaires are entered into the study website. Questionnaires returned by post to the trial office are entered there. The staff in the trial office work closely with local study teams to ensure the data is as complete and accurate as possible. The quality of data is enhanced by extensive range and consistency checks. Databases are backed up onto hard disc at an offsite location. All CRF and questionnaire keystrokes are recorded to maintain a full audit trail. All essential data and documents are retained for a period of at least 10 years after close of trial.

#### Confidentiality {27}

Data is stored on a secure database under the current Data Protection Legislation (General Data Protection Regulation and the Data Protection Act 2018). Personal data is not kept for longer than is necessary for the purpose for which it is processed. Access rights to the data set is managed.

#### Plans for collection, laboratory evaluation and storage of biological specimens for genetic or molecular analysis in this trial/future use {33}

Not applicable, no samples collected.

### Statistical methods

#### Statistical methods for primary and secondary outcomes {20a}

A statistical analysis plan will document the planned analysis, to be finalised before the data lock. All the main analyses will be based on the intention-to-treat principle. The final analysis will take place after full recruitment and follow-up. Baseline data will be summarised using the appropriate descriptive statistics and graphical summaries. A negative binomial regression of the number of blockages requiring intervention with the log of the number of days catheterised as an offset will be used to analyse the primary outcome. The regression will adjust for the minimisation covariates. A per-protocol analysis will be done as a sensitivity analysis. The appropriate generalised linear model will be chosen for the secondary outcomes and all models will adjust for the minimisation covariates.

#### Interim analyses {21b}

There are no planned interim analyses for efficacy or futility but an independent Data Monitoring Committee (DMC) will monitor trial progress and any safety issues.

#### Methods for additional analyses (e.g. subgroup analyses) {20b}

The following subgroup analyses are planned:

- Women vs men
- Neuropathic bladder vs. non-neuropathic bladder
- Age groups: < 45 vs. 45–64yrs vs. > 65
- Participants with no history of LTC blockages versus those with recurrent blockages
- Participants with no history of S-CAUTI vs those with recurrent S-CAUTI
- Participants with baseline urinary pH: normal range vs alkaline vs acidic

All subgroup analyses will be at the 99% significance level.

An economic evaluation is integrated into the trial and includes both a trial-based analysis and a modelling exercise to extrapolate the results over the patient’s lifetime. Outcomes and costs are assessed from the perspective of the NHS and patients. Effectiveness is measured in terms of quality-adjusted life years gained.

Qualitative interview and focus group transcripts will be analysed using an explicit, structured qualitative method of thematic analysis.

#### Methods in analysis to handle protocol non-adherence and any statistical methods to handle missing data {20c}

Data missing at baseline will be reported as such. If required, missing baseline primary and/or secondary outcome data will be imputed with centre specific mean for continuous data and missing binary/categorical data will include a missing indicator. Multiple imputation methods will be used for missing outcome data.

#### Plans to give access to the full protocol, participant-level data and statistical code {31c}

The full protocol is available on the NIHR website under award listing 17/30/02. Requests for participant-level data and/or statistical code can be made in writing to Professor Mohamed Abdel-fattah.

### Oversight and monitoring

#### Composition of the coordinating centre and trial steering committee {5d}

The trial office provides day-to-day support for the study sites and meets formally at least monthly. The trial manager takes responsibility for the day-to-day transaction of trial activities. The data coordinator provides clerical support to the trial.

The trial is supervised by its Project Management Group (PMG) which consists of the grant holders and representatives from the trial office, including the trial manager, data coordinator, statistician, health economist and qualitative researcher. The PMG meet at least quarterly throughout the study.

A Trial Steering Committee (TSC), with independent members, oversees the conduct and progress of the trial. The TSC meet at least annually throughout the study.

#### Composition of the data monitoring committee, its role and reporting structure {21a}

An independent Data Monitoring Committee (DMC) oversees the safety of subjects in the trial. The DMC comprises members with clinical, statistical and methodological expertise. The charter is filed in the TMF. The Committee meet at least annually throughout the study to monitor the trial data and make recommendations to the TSC.

#### Adverse event reporting and harms {22}

In CATHETER II, serious adverse events (SAEs) are recorded from each participant from the time a participant consents to joining the study until their last trial follow-up. The occurrence of SAEs is queried by the site at every contact with the participant or the person carrying out the washout. An event must be serious and related to the catheter or the catheter washout procedure to be considered an SAE in CATHETER II. In addition, all deaths by any cause are recorded as SAEs in CATHETER II. Adverse events captured as outcome measures (for example, catheter blockage) for the study are not reported through SAE processes. Any hospitalisation or prolongation of hospitalisation planned prior to randomisation, for elective treatment of a pre-existing condition or due to events captured as outcome measures are not recorded as SAEs. The investigator (or delegate) reviews appropriate documentation related to the SAE and records the details on the SAE form. If an SAE is recorded on a participant questionnaire, the trial office liaises with the clinical team to obtain further information if appropriate. The seriousness, relatedness and expectedness of the event are evaluated by the Investigator or the CI or delegate. If an event is confirmed as being a related and unexpected SAE, the trial office notifies the sponsor within 24 h and the CI (or delegate) reports any related and unexpected SAEs to the Research Ethics Committee (REC) within 15 days of the CI being aware of the event. All SAEs are regularly reported in progress reports to the REC, funder, DMC and TSC.

#### Frequency and plans for auditing trial conduct {23}

The trial office monitors oversight arrangements, training, set-up, data collection and safety as detailed in the study monitoring plan. The sponsor audits and monitors the trial and individual sites may have further arrangements locally for monitoring.

#### Plans for communicating important protocol amendments to relevant parties (e.g. trial participants, ethical committees) {25}

Protocol amendments require approval by the sponsors, funder, REC and the site. B. Braun Medical AG is notified of protocol amendments.

#### Dissemination plans {31a}

Trial results will be published in journals and presented at conferences and shared with relevant patient and clinical interest groups. A lay summary of the findings will be sent to participants.

## Discussion

Catheter care pathways are complex and heterogenous across the UK, with varying involvement and responsibilities for prescriptions, catheter changes and clinical management across GP practices, district and community nurses, secondary/tertiary care and social care. The CATHETER II study adopts multiple recruitment strategies to identify and recruit potentially eligible participants.

Recruitment to the study was temporarily paused at the onset of the COVID-19 pandemic. Adaptations made to the study protocol to limit face-to-face contact, minimise the risk to participants and research staff and resume recruitment included: the use of post and telephone for consent and baseline data collection; the training of participants and/or their carer to administer washouts by video consultation (where possible); the adoption of local/NHS infection control policy where face to face training to administer washouts is required; optional baseline urine dipstick pH test with participants provided with testing kits and instructions to self-perform the test; and pre-recorded webinars replacing site initiation visits. Follow-up proceeds by post and telephone as per the original protocol. At the time of submission of this publication, the study continues to be delivered in accordance with these COVID-19 pandemic adaptations.

A 2019 qualitative study [22] cited potential recruitment barriers in a hypothetical clinical trial comparing regular catheter washouts against standard care. One barrier is patients being unwilling to change their current catheter care if they are happy with how it is currently managed. The CATHETER II protocol permits changes to washout policy if clinically necessary. Another cited recruitment barrier is the complex health issues/co-morbidities in the study population with these patients often not having time to commit to research. CATHETER II minimises face-to-face clinical visits, with nearly all study activities delivered remotely by post, phone and videocall. Feedback from sites to date indicates that these barriers remain an issue for a proportion of potential participants.

No other robust randomised controlled trials investigating the study question are published or in progress. The research question remains a very important clinical dilemma for patients, clinicians, nursing organisations and NHS policymakers.

## Trial status

Recruitment commenced on 12 December 2019. Recruitment to the trial was paused on 16 March 2020 due to the COVID-19 pandemic and re-opened to recruitment in September 2020. Due to ongoing delays as a result of the COVID-19 pandemic, it is not possible to provide an approximate end date for the recruitment. The current protocol is version 10, 07 March 2022.

## Supplementary Information

**Additional file 1.** CATHETER II full participant patient information leaflet.

**Additional file 2.** CATHETER II participant consent form.

## Data Availability

The trial statistician and health economist will have access to the full dataset to permit analysis at the end of the trial. The final trial dataset generated and/or analysed during the current study may be available on reasonable request to the CI.

## Abbreviations

CRF: Case report form
DMC: Data Monitoring Committee
EQ-5D-5L: EuroQol Questionnaire – 5 Dimensions – 5 Levels
GCP: Good Clinical Practice
GP: General practitioner, general practice
GSE: General Self-Efficacy Scale
ICECAP-A: ICEpop CAPability measure for Adults
ICECAP-O: ICEpop CAPability measure for Older people
ICIQ-LTCqol: International Consultation on Incontinence Modular Questionnaire – Long-Term Catheter quality of life
LTC: Long-term catheter
NICE: National Institute for Health and Clinical Excellence
NIHR HTA: National Institute of Health Research Health Technology Assessment Programme
NRS: NHS Research Scotland
PMG: Project Monitoring Group
REC: Research Ethics Committee
SAE: Serious adverse event
S-CAUTI: Symptomatic catheter-associated urinary tract infection
TSC: Trial Steering Committee
UK: United Kingdom
