# Supplementary material for: Randomised controlled trial comparing the clinical and cost-effectiveness of various washout policies versus no washout policy in preventing catheter associated complications in adults living with long-term catheters: study protocol for the CATHETER II study
Source: Trials. 2022 Aug 4;23:630. doi: 10.1186/s13063-022-06577-2 (PMC9351274; doi:10.1186/s13063-022-06577-2)
Supplement: Supplementary file 1 — Additional file 1. CATHETER II full participant patient information leaflet. [file 13063_2022_6577_MOESM1_ESM.pdf]

PATIENT INFORMATION LEAFLET

The CATHETER II study

INVITATION TO TAKE PART

We are trying to find out the best way to reduce the number of blockages that can happen in people who have long term indwelling catheters and we would like to ask you to help.

We are carrying out a study funded by the National Institute of Health Research, (the research arm of the NHS), to find out if washing out the catheter every week using catheter washout liquids reduces catheter blockages and other problems like urinary incontinence or urinary tract infections.

We are inviting you to take part because you use the type of catheter we are studying.

We aim to recruit 600 people from all over the UK who use the same type of catheter. Each person who joins the study will be in the study for a maximum of 2 years.

Before you decide, it is important that you understand why the research is being done and what it will involve so please take time to read this leaflet carefully. Please talk to other people about taking part, people like your GP or members of your family and ask us if there is anything that is not clear or if you want more information.

BACKGROUND TO THE CONDITION

Urinary catheters are soft tubes put into the bladder to drain and then collect urine. In the UK, an average of 1 in 1000 people use long-term indwelling catheters like you do.

People using these catheters can experience complications, like blockages (where the urine does not drain into the catheter bag properly); urinary infections; pain and a type of incontinence called catheter bypass. These complications can affect a person's quality of life. They may also mean more emergency visits with nurses and GPs.

Our research shows that people consider blockage to be one of the most troubling aspects of using catheters over a long period. In current standard catheter care, catheter blockages are dealt with by either changing the catheter more often and/or using catheter washout liquids to washout the catheter.

Some people are advised to do a catheter washout every week to try to reduce blockages. Others are not. The difference is because at present, there is no clear evidence to show whether doing regular washouts helps avoid blockages.

People doing washouts also use different solutions. One is a weak salty solution, and the other is a citric mix, more like

weak lemon juice. Both solutions are used in the NHS but we do not know which works best.

WHAT IS THE PURPOSE OF THE STUDY?

Doctors, nurses and patient groups representing people with long-term catheters agree that a study is needed to decide if catheter washouts help prevent blockages. That is what this study hopes to do.

We will be looking to see if weekly catheter washout solutions can prevent blockage and improve the quality of life for patients. We also want to find out if one type of washout liquid works better than the other. We will also study which treatment makes best use of NHS resources.

In the CATHETER II study we are comparing three groups of patients:

1) those receiving standard catheter care only (with no weekly washouts)

2) those receiving standard catheter care plus weekly washing out of the catheter with saline (weak salty) solution

3) those receiving standard catheter care plus weekly washing out of the catheter with citric (like a weak lemon juice) solution.

DO I HAVE TO TAKE PART?

No. It is entirely up to you whether or not you take part. Please take as much time as you need to decide. Read this leaflet, ask the research team, your doctor, nurse, family members, and/or carer(s) as many questions as you like.

If you do decide to take part you will be asked to sign a form giving your consent to be included in the study.

You can change your mind at any time and withdraw from the study. Whatever decision you make will not affect the standard of care you receive now or in the future.

If you say yes but then decide to withdraw we will keep the information we have already collected about you for the study results. This information will remain confidential and will not be used for any other purpose. To safeguard your rights, we will use the minimum personally-identifiable information possible.

WHAT WOULD TAKING PART INVOLVE?

If you decide to take part, we will ask you to complete a questionnaire about your quality of life, your catheter and any problems you have with it. We will also ask you for a urine sample.

A computer will then be used to decide at random which of the three groups outlined above you will join. You will not be able to choose which group you join. You will be asked to take part in the study for 2 years.

Whichever group you are in, you will still continue to receive the current standard NHS long term catheter care, which includes a written plan. The plan gives information about drinking enough; hand and catheter hygiene; how to secure the catheter position; how to avoid catheter kinking; how often to change the catheter and the catheter bag/ valve; and advice on how to prevent and manage problems. The plan will include the contact details for your healthcare team

in case you want to discuss anything about your care or if you experience problems related to your catheter.

If you are put in one of the study groups doing the weekly catheter washouts, a nurse will show you (and/or your relative, friend or other person) how to carry out the washouts correctly. You will also be given a detailed, illustrated leaflet showing you how to do the washout. The washout solutions are free and will be sent to you at your home.

Most people find using a washout solution quite simple. The washout liquid comes in a prefilled sealed bag (2 x 30mls – about the same volume as 4 tablespoons, or 100ml – about the same volume as half a cup). At the same time as the routine weekly change of the catheter bag/ valve you connect the washout solution bag to the catheter valve and introduce the washout liquid into the bladder with the help of gravity. If you are using the saline solution, you drain it back into the same bag with the help of gravity. If you are using the citric solution, you leave the liquid in the bladder for 5 minutes and then drain it back into the same bag with the help of gravity, which is then repeated.

We will follow you in the study for 2 years. We will give you a special calendar where we would like you to record any problems with your catheter. You will also be contacted each month by a member of the research team who will ask about any catheter-related problems you may have had.

Every six months, we will ask you to complete a questionnaire about your quality of life and your satisfaction with treatment. We will remind you once about completing the questionnaires. If you need help from a relative, friend or other person to complete the calendar, the

questionnaires or to use the catheter washouts we will ask you for their contact details so we can discuss the study with them.

When we receive your completed questionnaires, we will send you a shopping voucher to thank you for your help. If you don't want to receive this, please let us know on the consent form.

At the end of the study, with your permission, we may ask your GP, community or district nurse if they can tell us about any catheter problems that you have had since you joined the study. If you live in a care home we would ask the care home manager if they can tell us this information.

There is an extra part to the CATHETER II study that is optional. Some people will be asked to take part in an interview. We will ask you whether or not you would like information about these interviews if you decide to take part in the study.

In the next 10 years, we may want to carry out further research studies with people who have a long-term catheter. We would like to ask your permission to contact you about these research studies. This also is optional. You can take part in the CATHETER II study without agreeing to be contacted about further research studies.

WHAT ARE THE POSSIBLE BENEFITS OF TAKING PART?

You will receive the same level of care from your healthcare team whether or not you choose to take part in the study. You may not benefit personally from taking part, but you will

be directly helping us to improve the care of patients with a long-term catheter in the future.

WHAT ARE THE DISADVANTAGES, RISKS AND SIDE EFFECTS OF REGULAR CATHETER WASHOUTS?

The washout solutions that we are using in the study are already being used in the NHS.

There may be a possible increase in risk of urine infection when doing regular catheter washouts. We will monitor this closely within the study and we will ask you about urine infections during every follow-up in the study.

There are some side-effects from the long-term use of catheters but we do not think that the risk will be increased by introducing regular washouts.

WHAT HAPPENS WHEN THE RESEARCH STUDY STOPS?

You will continue your care and treatment in line with NHS standard clinical care. If you are part of one of the groups doing weekly catheter washouts, we will not be able to send you any more solutions at the end of the study.

If the study is stopped earlier than expected for any reason, we will tell you and ensure that your continuing care is arranged.

WHAT IF THERE IS A PROBLEM?

We do not expect any harm to come to you by taking part in this study.

If you believe that you are harmed by taking part you can complain and seek compensation through the research

sponsors of this study, the University of Aberdeen and NHS Grampian.

If you wish to complain or have any concerns about any aspect of the way you have been approached or treated during the course of this study, the normal NHS complaints mechanisms would be available to you.

If you are concerned about any aspect of the study, you can speak with the research team who will do their best to answer your questions. Contact details of your local study nurse and the Study Office can be found at the end of this information sheet.

You can also contact the chair of the Trial Steering Committee, who is independent from the study, through the study office. If you remain unhappy and wish to complain formally, you can do this through the NHS Complaints Procedure.

<<*Additional information for participants in England and Wales:* The Patient Advice and Liaison Service (PALS) offers confidential advice, support and information for health-related matters. They provide a point of contact for patients, their families and their carers. Your local PALS details are given at the end of this information sheet.>>

<<*Additional information for participants in Scotland:* NHS Health Scotland is the national Health Board working to reduce health inequalities and improve population health in Scotland. Further contact information is given at the end of this information sheet.>>

WILL MY TAKING PART IN THE STUDY BE KEPT CONFIDENTIAL?

Yes. We will follow ethical and legal practice and all information collected about you for the purpose of this research study will be handled in strict confidence and securely stored by the University of Aberdeen.

WHO WILL HAVE ACCESS TO MY INFORMATION?

The local research team at the hospital, GP practice or care home that you were recruited in will have access to your information. They will use your name and contact details to contact you about the study, to make sure that relevant information about the study is recorded for your care, and to oversee the quality of the study.

We, ***the study team***, who are based in the Centre for Healthcare Randomised Trials (CHaRT) at the University of Aberdeen, will have access to your information to contact you about the study, for example to send you the questionnaires, and for quality control purposes, such as auditing the data collection processes. All electronic data collected for the purpose of the research study will be confidentially and securely stored on computer servers maintained by the University of Aberdeen. The local research team will pass information collected from you and your medical records to the study team.

With your permission, we will pass on your name and address to a courier company to deliver the washout solutions to your home. These details will be kept strictly confidential and be used only to send you the washout solutions.

We will tell your GP you are taking part, but only with your permission.

All data for the statistical analysis of the study is being conducted at the University of Aberdeen and will be anonymous. Your name and address will not be included, and it will not be possible to identify individuals. Any reports or publications arising from the study will also contain only anonymous data.

Other individuals from the University of Aberdeen and NHS Grampian, the Research and Development Department of your local NHS Organisation and the Regulatory Authorities may look at your medical records and data collected for the study, to check that the study is being carried out correctly and to check the accuracy of the research study. All will have a duty of confidentiality to you as a research participant.

Other researchers may wish to access anonymous data from this study for future research. If this is the case, they would be expected to follow legal, data protection and ethical guidelines. It will not be possible to identify you from this data. The information will only be used for the purpose of health and care research and cannot be used to contact you or affect your care.

Your rights to access, change or move your information are limited, as we need to manage your information in specific ways in order for the research to be reliable and accurate.

WHAT IF RELEVANT NEW INFORMATION BECOMES AVAILABLE?

If new information becomes available that is relevant to the project the CATHETER II Study team will contact you to let you know about the choices available to you.

HOW LONG WILL MY INFORMATION BE KEPT?

All information which is collected about you during the research, including identifiable data, will be held securely for 10 years after the trial has finished in accordance with Sponsor requirements and data legislation.

WHO IS RESPONSIBLE FOR MY INFORMATION?

The University of Aberdeen is the controller for this study and is responsible for looking after your information, using it properly and complying with your rights. You can find more about this at www.abdn.ac.uk/privacy or by contacting us at the address below.

WHAT WILL HAPPEN TO THE RESULTS OF THE STUDY?

The results of the study will be used to make recommendations on care for patients who have long-term catheters. We shall publish the results of this study in scientific journals and present the information at appropriate meetings. You will not be identified in any publication of results of the study. We will let you know the results of the study when it is finished unless you tell us that you do not wish to know.

WHO IS ORGANISING AND FUNDING THE STUDY?

This study is sponsored by the University of Aberdeen and NHS Grampian who have overall responsibility for the

management of the study. The study is funded by the National Institute of Health Research (the research arm of the NHS). The research is being carried out by a group of experienced doctors, nurses, and researchers in collaboration with the Centre for Healthcare Randomised Trials (CHaRT), a UKCRC registered Clinical Trials Unit at the University of Aberdeen.

Several organisations are involved and are supporting this study including the Royal College of Nursing, Ageing UK, and British Association of Urology nurses.

WHO HAS REVIEWED THE STUDY?

All research in the NHS is looked at by an independent group of people, called a Research Ethics Committee (REC) to protect your interests. This study has been reviewed and given favourable opinion by Wales Research Ethics Committee 6.

Thank you for reading this

Thank you for taking the time to read this information leaflet. We hope that it has been helpful in enabling you to decide if you would like to participate in the Catheter II study. Please ask us if you have questions or would like more information about the study.

FURTHER INFORMATION AND CONTACT DETAILS

If you have any questions or would like any more information, please contact:

**CATHETER II Trial Office<br>Centre for Healthcare Randomised Trials (CHaRT), Health Services Research Unit<br>University of Aberdeen<br>Health Sciences Building<br>Foresterhill<br>Aberdeen AB25 2ZD<br>Tel: 01224 [redacted]<br>Fax: 01224 [redacted]<br>Email: [redacted]<br>Web: w3.abdn.ac.uk/hsru/CatheterII/**	**<<*To be entered for each site:*<br><br>*Local centre contact details*<br><br>PALS contact information (England and Wales) OR<br><br>NHS Health Scotland, <<website address>> and <<contact address>><br><br>>>**

FUNDED BY

NIHR | National Institute for Health Research

CATHETER II Study is funded by the National Institute for Health Research Health Technology Assessment Programme (NIHR HTA)
