# Supplementary material for: Randomised controlled trial comparing the clinical and cost-effectiveness of various washout policies versus no washout policy in preventing catheter associated complications in adults living with long-term catheters: study protocol for the CATHETER II study
Source: Trials. 2022 Aug 4;23:630. doi: 10.1186/s13063-022-06577-2 (PMC9351274; doi:10.1186/s13063-022-06577-2)
Supplement: Supplementary file 2 — Additional file 2. CATHETER II participant consent form. [file 13063_2022_6577_MOESM2_ESM.pdf]

Participant Study Number

TRIAL CONSENT FORM
CATHETER II

IRAS 259559

CATHETER II

Please INITIAL all boxes

By INITIALLING each box and signing this form:

1) I confirm that I have

read the Information Sheet about the CATHETER II study (Version number ……., date ………………………..). I have had the opportunity to consider the information, ask questions and have had these answered satisfactorily.

2) I understand that

a) my participation is voluntary and that I am free to withdraw at any time without giving any reason, without my medical care or legal rights being affected. Data collected up until the point of withdrawal may still be used in analysis.

b) relevant sections of my medical and care home notes and data collected during the trial may be looked at by individuals directly involved in the trial, from the University of Aberdeen or from the NHS Boards or Trusts, where it is relevant to my taking part in this research. I give permission for these individuals to have access to them.

c) relevant data collected for the purpose of the trial together with personal contact details will be confidentially and securely stored by the University of Aberdeen in accordance with data protection legislation/regulations. I agree that the study co-ordinators can use my contact details to send me study questionnaires and to contact me by <<phone or post or email or text>>.
I agree my contact details will be shared with the company that will deliver the washout solutions (if I am in the washout group) to my home.

d) the information collected about me may be shared anonymously with other researchers to support future research.

I agree to take part in the study

OPTIONAL

I agree to my General Practitioner being informed of my participation in this study

I agree to be contacted for possible participation in the interview study

I agree to receive a token of appreciation by post

I am willing to be contacted in the future about participating in other relevant research.

Your signature (participant)	Name in BLOCK capitals	Date

To be completed by the local team member taking consent

I confirm that I have explained to the person named above, the nature and purpose of the study and the procedures involved.

Signature	Name in BLOCK capitals	Date

CATHETER II Trial Office, Centre for Healthcare Randomised Trials (CHaRT), Health Services Research Unit, University of Aberdeen, Scotland AB25 2ZD; Tel 01224 [redacted]; Fax 01224 [redacted]; Email [redacted] **Copies:** Original for research site file; 1 for participant (carbon copy); 1 to be kept in medical notes (copy); 1 for trial office (copy).

CATHETER II Study is funded by the National Institute for Health Research Health Technology Assessment Programme (NIHR HTA)
